# Rapid Dilatation of the Uterus, for Hemorrhage

**Published:** 1892-07

**Authors:** 


RAPID DILATATION OF THE UTERUS, FOR THE TREATMENT OF HEMORRHAGE.—In a paper read before the Medical Society of London, March 28, 1892, Dr. A. Routh presented the following conclusions:

1. Menorrhagia, and especially metrorrhagia, constitute an indication for dilatation.
2. This is best done by graduated bougies under an anæsthetic.
3. Pyrexia is not to be feared, if the procedure is carried out under antiseptic precautions, unless malignant disease or tubal disease is present.
4. That even with tubal disease, dilatation is not necessarily contra-indicated.
5. That dilatation should precede all other operations for the relief of hemorrhage.
6. That dilatation often suffices to effect a cure.—*Medical Press and Circular*, March 30, 1892.

